# A Pre-Trained Fuzzy Reinforcement Learning Method for the Pursuing Satellite in a One-to-One Game in Space

**DOI:** 10.3390/s20082253

**Published:** 2020-04-16

**Authors:** Xiao Wang, Peng Shi, Yushan Zhao, Yue Sun

**Affiliations:** 1 School of Astronautics, Beihang University, Beijing 100191, China; w_xiao@buaa.edu.cn (X.W.); yszhao@buaa.edu.cn (Y.Z.); 2 Shanghai Key Laboratory of Aerospace Intelligent Control Technology, Shanghai Aerospace Control Technology Institute, Shanghai 201109, China; yoyo_326@163.com

**Keywords:** differential game, reinforcement learning, actor-critic, fuzzy system

## Abstract

In order to help the pursuer find its advantaged control policy in a one-to-one game in space, this paper proposes an innovative pre-trained fuzzy reinforcement learning algorithm, which is conducted in the *x*, *y*, and *z* channels separately. Compared with the previous algorithms applied in ground games, this is the first time reinforcement learning has been introduced to help the pursuer in space optimize its control policy. The known part of the environment is utilized to help the pursuer pre-train its consequent set before learning. An actor-critic framework is built in each moving channel of the pursuer. The consequent set of the pursuer is updated through the gradient descent method in fuzzy inference systems. The numerical experimental results validate the effectiveness of the proposed algorithm in improving the game ability of the pursuer.

## 1. Introduction

Tracking space targets is beneficial for orbital garbage removal, recovery of important components, and early warning of space threats [1]. However, with the continuous development of space techniques, the targets in space have been expanded from non-maneuverable ones to maneuverable ones. Tracking a target that has maneuverability is still a challenging problem because the target is non-cooperative and the environment is usually partially unknown.

In order to track a non-cooperative target in space, one can apply control theory to design a control law. By establishing an attitude-position coupling model, an adaptive control law that considers the unknown mass and inertia was proposed [2]. Besides considering the system static errors and disturbances, some adaptive control laws were designed [3,4]. With the development of the research, many mature control methods were introduced to the field of tracking targets in space [5,6,7]. In addition, a back-stepping adaptive control law with the consideration of a variety of model uncertainties, as well as the input constraints and an optimal inverse controller with external disturbances were attempted in [8,9], respectively. It was proven that the closed-loop system was still stable in the presence of external disturbances and uncertain parameters. However, these proposed control laws are basically used for the targets, which do not have the ability to maneuver.

For tracking a target that can move, there is the potential to describe the problem as a pursuit-evasion problem, which is also known as the space differential game. The differential game, which was introduced in [10,11], is usually applied to continuous systems. To find a superior strategy of the pursuer in aircraft combat, scholars proposed the proportional navigation method [12]. In addition, when the differential game is applied to the field of space, the so-called two-sided optimal theory, which was an extension of the traditional optimal theory, is found in [13,14]. Besides, in order to transform the two-sided optimal problem into a traditional one-sided optimal problem, the semi-direct collocation method was studied based on the two-sided extremum principle [15]. In order to reduce the difficulty of the solution, the genetic algorithm was employed to help find the initial values of the co-states [16,17,18]. However, based on the two-sided optimal theory, the optimal strategy of the pursuer can only be found when the system information is totally known, and it will be unable to deal with system uncertainties and external disturbances. Therefore, it is reasonable to find a way to make the pursuer able to adjust its control policy according to the environment. One of the potential methods is to apply reinforcement learning because of its capacity to optimize the control policy under an unknown environment.

Reinforcement learning, which aims to map states to actions so as to maximize a numerical reward, is one of the machine learning methods [19]. At first, reinforcement learning was used for solving problems of discrete systems after the classical Q-learning was proposed [20], and this branch has been developed [21]. Since the technique of space generalization was introduced to avoid the curse of dimensionality, the learning algorithms can be applied to solve the problems in continuous space [22,23,24,25]. As for solving a differential game, in recent years, scholars have also found that it was effective to use the reinforcement learning algorithm [26,27]. With the single control input, the ground pursuit-evasion problem was considered in [28,29].

There seems to be potential in using the technique of reinforcement learning because such a learning method can help the pursuer optimize its control policy in an unknown environment. However, the differential game in space has more complex dynamics; therefore, it will be extremely hard to solve without any prior information. To overcome this shortcoming, in this paper, we propose an innovative pre-trained fuzzy reinforcement learning (PTFRL) algorithm to help the pursuer optimize its control policy through a pre-training process. The pre-training process utilizes the known part of the environment and helps the pursuer initialize its consequent set before reinforcement learning. The algorithm is based on the actor-critic framework, which is one of the most active reinforcement learning branches. The learning framework is divided into *x*, *y*, and *z* channels, and each channel learns separately. The man-made model is defined as an estimated environment, which can be used to derive the estimated optimal strategy for the pursuer. With the help of the genetic technique, the pre-training process will be conducted to help the pursuer initialize its consequent set. Then, through the fuzzy inference systems, the control policy of the pursuer will be updated from the fuzzy actor-critic learning.

In general, this paper applies a pre-trained fuzzy reinforcement learning algorithm to optimize the control policy of a pursuer, which is used for a one-to-one game in outer space. The main improvements of this paper are as follows: (1) Unlike the previous control laws, which were designed based on the adaptive control theory, for the first time, we utilize the technique of reinforcement learning to help the pursuer track a moving non-cooperative target in space. Compared with the adaptive control laws, which contain massive derivations and computing costs to deal with the uncertainties of the environment, the proposed algorithm takes advantage of artificial intelligence, avoiding the mathematical complexity. It is a new approach to optimize the control policy of the pursuer by interacting with the space environment. (2) Different from the reinforcement learning algorithms applied in ground games, the game in space has more complex states and actions. Without any prior information, it will be extremely hard for the pursuer to find its control policy because of the complex environment. To reduce the difficulty of solving the game, the proposed algorithm innovatively adds a pre-training process utilizing the known part of the environment.

The structure of this paper is as follows: Section 2 presents the dynamics of the pursuer and the evader; Section 3 discusses the fuzzy inference system and its combination with reinforcement learning for continuous systems; Section 4 applies the pre-trained fuzzy reinforcement learning algorithm for the pursuer; Section 5 simulates the proposed algorithm; Section 6 discusses the experimental results; finally, Section 7 draws the conclusions.

## 2. Dynamics of the Space Differential Game

To describe the space differential game, the following coordinate systems are established: (a) Earth-centered inertial (OXYZ); (b) the orbital coordinate system of the spacecraft ($Ox_{o}y_{o}z_{o}$); (c) the orbital coordinate system of the virtual host spacecraft ($Ox_{r}y_{r}z_{r}$).

**Figure 1:**
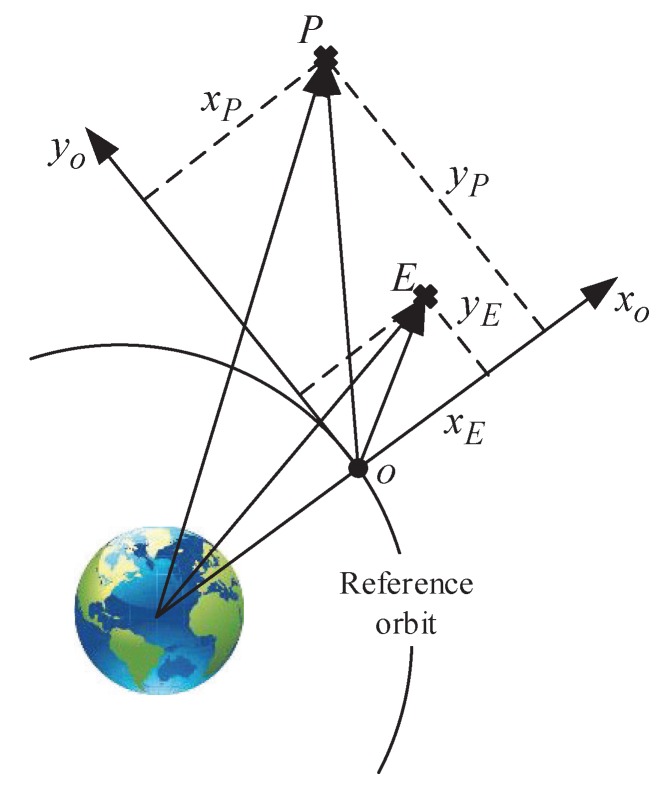
The location of the pursuer and the evader.

In this game, there are one pursuer and one evader, where the pursuer *P* aims to track the evader *E* and the evader *E* aims to escape from the pursuer *P*. The position relationship among the pursuer, the evader, and the virtual host point *o* is drawn in Figure 1.

The virtual host point *o* is located near the two satellites. The pursuer and the evader can be abstracted as the agents, which have the ability of interacting with the environment. In this paper, we will focus on the control strategy of the pursuer to make it have an advantage in this game. The pursuer is expected to update its control policy according to its interaction with the environment through reinforcement learning. Therefore, for a simulated experiment in this paper, it is necessary to build an environment that includes the dynamics of the agents in it.

This pursuit-evasion game is supposed to occur in the neighborhood of a near circular reference orbit. In addition, it is supposed that there may exist an external disturbance force acting on the pursuer and the evader. Denote the position of satellite *P* as $x_{P}={\left[x_{P},y_{P},z_{P}\right]}^{T}$, while the position of satellite *E* as $x_{E}={\left[x_{E},y_{E},z_{E}\right]}^{T}$. Therefore, the dynamics of the pursuer, *P*, is expressed as below [30]:

(1) $$\left\{\begin{array}{cc} \; & {\dot{x}}_{P}\left(t\right)=v_{P}^{x}\left(t\right)\\ \; & {\dot{y}}_{P}\left(t\right)=v_{P}^{y}\left(t\right)\\ \; & {\dot{z}}_{P}\left(t\right)=v_{P}^{z}\left(t\right)\\ \; & {\dot{v}}_{P}^{x}\left(t\right)=2\frac{\mu }{r^{3}\left(t\right)}x_{P}\left(t\right)+2\omega \left(t\right)v_{P}^{y}\left(t\right)+\dot{\omega }\left(t\right)y_{P}\left(t\right)+\omega^{2}\left(t\right)x_{P}\left(t\right)+T_{P}u_{P}^{x}\left(t\right)+d_{t}^{x}\\ \; & {\dot{v}}_{P}^{y}\left(t\right)=-\frac{\mu }{r^{3}\left(t\right)}y_{P}\left(t\right)-2\omega \left(t\right)v_{P}^{x}\left(t\right)-\dot{\omega }\left(t\right)x_{P}\left(t\right)+\omega^{2}\left(t\right)y_{P}\left(t\right)+T_{P}u_{P}^{y}\left(t\right)+d_{t}^{y}\\ \; & {\dot{v}}_{P}^{z}\left(t\right)=-\omega^{2}\left(t\right)z_{P}\left(t\right)+T_{P}u_{P}^{z}\left(t\right)+d_{t}^{z} \end{array}\right.$$

where μ represents the Earth’s gravitational constant, $\omega \left(t\right)$ represents the instantaneous angular velocity of the reference orbit, and $r\left(t\right)$ represents the instantaneous radius of the orbit. Besides, the dynamics of the evader *E* is expressed as follows.

(2) $$\left\{\begin{array}{cc} \; & {\dot{x}}_{E}\left(t\right)=v_{E}^{x}\left(t\right)\\ \; & {\dot{y}}_{E}\left(t\right)=v_{E}^{y}\left(t\right)\\ \; & {\dot{z}}_{E}\left(t\right)=v_{E}^{z}\left(t\right)\\ \; & {\dot{v}}_{E}^{x}\left(t\right)=2\frac{\mu }{r^{3}\left(t\right)}x_{E}\left(t\right)+2\omega \left(t\right)v_{E}^{y}\left(t\right)+\dot{\omega }\left(t\right)y_{E}\left(t\right)+\omega^{2}\left(t\right)x_{E}\left(t\right)+T_{E}u_{E}^{x}\left(t\right)\\ \; & {\dot{v}}_{E}^{y}\left(t\right)=-\frac{\mu }{r^{3}\left(t\right)}y_{E}\left(t\right)-2\omega \left(t\right)v_{E}^{x}\left(t\right)-\dot{\omega }\left(t\right)x_{E}\left(t\right)+\omega^{2}\left(t\right)y_{E}\left(t\right)+T_{E}u_{E}^{y}\left(t\right)\\ \; & {\dot{v}}_{E}^{z}\left(t\right)=-\omega^{2}\left(t\right)z_{E}\left(t\right)+T_{E}u_{E}^{z}\left(t\right) \end{array}\right.$$

where $u_{i}^{j}$(j=x,y,z) represents the force in the corresponding channel and $T_{i}$(i=P,E) represents the maximum unit mass thrust of the satellite. It is noted that the external disturbance force is only added to the pursuer, because we always consider the relative states between the pursuer and the evader.

Through Equations (1) and (2), the environment for the learning algorithm is built, and it is seen as the real environment, which is differentiated from the estimated environment referred to in Section 4.2.

## 3. Reinforcement Learning in Continuous Systems

To avoid the curse of dimensionality, the technique of generalization should be addressed. Besides, the problem regarding satellite motion requires the inputs of the learning system to have clear physical meaning. Therefore, the zero-order Takagi–Sugeno (T-S) fuzzy system, which provides a more meaningful inference rule compared with neural networks, is employed as the approximator. In this way, the fuzzy actor-critic learning framework will be built. Through the gradient descent method, the consequent parameters of the actor and the critic will be updated.

### 3.1. The Fuzzy Inference System

The fuzzy inference rule of the employed Takagi–Sugeno (T-S) fuzzy system is expressed as below [31].

(3) $$Rule\;l:\mathrm{IF}\;s_{1}\;is\;F_{1}^{l},\cdots ,\;and\;s_{n}\;is\;F_{n}^{l}\;\;\;\;THEN\;z_{l}=\phi_{l}$$

If we assume that the fuzzy system has *L* rules, *n* input variables, and each input has *h* membership functions, the output of the fuzzy system can be expressed as:

(4) $$Z\left(s\right)=\frac{\sum_{l=1}^{L}\left[\left(\prod_{i=1}^{n}\mu^{F_{i}^{l}}\left(s_{i}\right)\right)\phi^{l}\right]}{\sum_{l=1}^{L}\left(\prod_{i=1}^{n}\mu^{F_{i}^{l}}\left(s_{i}\right)\right)}=\sum_{l=1}^{L}\Psi_{l}\left(s\right)\phi_{l}$$

where $s_{i}\left(i=1,\cdots ,n\right)$ represents the ith input of the fuzzy system, $F_{i}^{l}$ represents the fuzzy set of the ith input variable, $z_{l}$ represents the output of the lth rule, $\phi_{l}$ represents the consequent parameter, $s={\left[s_{1},\cdots ,s_{n}\right]}^{T}$ represents the state vector, and $\mu^{F_{i}^{l}}$ represents the membership function of $s_{i}$ under the lth rule. The expression of $\Psi_{l}\left(s\right)$ is as follows.

(5) $$\Psi_{l}\left(s\right)=\frac{\prod_{i=1}^{n}\mu^{F_{i}^{l}}\left(s_{i}\right)}{\sum_{l=1}^{L}\left(\prod_{i=1}^{n}\mu^{F_{i}^{l}}\left(s_{i}\right)\right)}=\frac{\omega_{l}\left(s\right)}{\sum_{l=1}^{L}\omega_{l}\left(s\right)}$$

**Figure 2:**
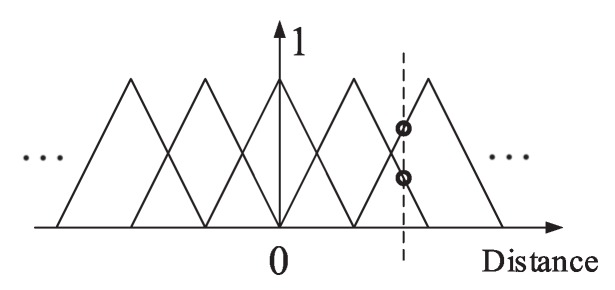
The membership functions for one input.

The applied membership functions here are triangular membership functions, which are shown in Figure 2. This shows that the input will only activate two membership functions at one time for one input, which will save computing cost when the number of membership functions rises.

### 3.2. The Fuzzy Actor-Critic Learning Algorithm

In the actor-critic learning algorithm, the value function and the policy function are approximated through T-S systems, respectively. The critic part is used to estimate the value function, while the actor part is used to generate the action. To apply the actor-critic learning framework into a continuous system, we need two critic parts to estimate the current value function ${\hat{V}}_{t}\left(s_{t}\right)$ and the next value function ${\hat{V}}_{t}\left(s_{t+1}\right)$ and one actor part to generate the current control variable. In this way, the temporal difference can be expressed as below.

(6) $$\Delta_{t}=r_{t}+\gamma {\hat{V}}_{t}\left(s_{t+1}\right)-{\hat{V}}_{t}\left(s_{t}\right)$$

Denote $\Xi =\frac{1}{2}\Delta_{t}^{2}$ as the variance of the difference signal; therefore, the adaptive update rule of the parameters in the critic is expressed as:

(7) $$\begin{array}{cc} \phi^{C}\left(t+1\right) & =\phi^{C}\left(t\right)-\alpha \frac{\partial \Xi }{\partial \phi^{C}}\\ \; & =\phi^{C}\left(t\right)-\alpha \Delta_{t}\left[\gamma \frac{\partial V_{t}\left(s_{t+1}\right)}{\partial \phi^{C}}-\frac{\partial V_{t}\left(s_{t}\right)}{\partial \phi^{C}}\right] \end{array}$$

where $\phi^{C}$ represents the consequent parameter of the critic and α represents the learning rate of the critic.

In addition, we have:

(8) $$\frac{\partial V_{t}\left(s_{t}\right)}{\partial \phi^{C}}=\left[\Psi_{1}\left(s_{t}\right),\Psi_{2}\left(s_{t}\right),\cdots ,\Psi_{L}\left(s_{t}\right)\right]$$

(9) $$\frac{\partial V_{t}\left(s_{t+1}\right)}{\partial \phi^{C}}=\left[\Psi_{1}\left(s_{t+1}\right),\Psi_{2}\left(s_{t+1}\right),\cdots ,\Psi_{L}\left(s_{t+1}\right)\right]$$

which can be combined with Equation (5). In this way, Equation (7) can be solved.

Denoting the output of the actor as $u_{t}$, a rand noise, σ, will be added to $u_{t}$ to explore better rewards. Therefore, the real output is $u_{c}=u_{t}+\sigma$.

Further, the adaptive update rule of the parameters of the actor is expressed as:

(10) $$\phi^{A}\left(t+1\right)=\phi^{A}\left(t\right)+\beta \Delta_{t}\frac{\partial u_{t}}{\partial \phi^{A}}\left(u_{c}-u_{t}\right)$$

where $\phi^{A}$ represents the consequent parameter of the actor and β represents the learning rate of the actor.

## 4. Pre-Trained Fuzzy Reinforcement Learning for the Pursuing Satellite in a One-to-One Game in Space

The proposed algorithm is single-looped, which means that for the motions of the pursuing satellite *P*, each agent has to be divided into three channels, the *x*, *y*, and *z* channels. In each channel, there exists two inputs, the relative distance and the relative velocity of the current channel. With the help of the genetic algorithm, the consequent sets of actors in each channel will be initialized.

### 4.1. Fuzzy Reinforcement Learning Algorithm

Take the *x* channel as an example. The inputs are $s_{1}=x$ and $s_{2}=v_{x}$; therefore, the inference rule is expressed as:

(11) $$R_{l}:\mathrm{IF}\;s_{1}\;is\;A_{1}^{l}\;and\;s_{2}\;is\;A_{2}^{l}\;\;THEN\;Z_{l}=\varphi_{l}$$

where $\varphi_{l}$ represents the consequent parameter in the consequent set $\varphi_{P}^{x}$ of critics.

In addition, the following relationship is shown.

(12) $$\Psi_{l}\left(s\right)=\frac{\prod_{i=1}^{2}\mu^{F_{i}^{l}}\left(s_{i}\right)}{\sum_{l=1}^{4}\left(\prod_{i=1}^{2}\mu^{F_{i}^{l}}\left(s_{i}\right)\right)}=\frac{\omega_{l}\left(s\right)}{\sum_{l=1}^{4}\omega_{l}\left(s\right)}$$

(13) $${\hat{V}}_{P}^{x}=\sum_{l=1}^{4}\left(\Psi_{l}\right)\cdot \left(\varphi_{l}\right)$$

Similarly, the output of the actor is shown as below.

(14) $$u_{t}=\sum_{l=1}^{4}\left(\Psi_{l}\right)\cdot \left(\phi_{l}\right)$$

where $\phi_{l}$ represents the consequent parameter in the consequent set $\phi_{P}^{x}$ of actors. To add a noise σ for exploring, the final control variable is expressed as follows.

(15) $$u_{P}^{x}=u_{t}+\sigma$$

The designed reward function, $r_{t}$, is expressed as:

(16) $$\begin{array}{cc} \; & r_{t}|{}_{P}^{x}=D_{x}\left(t-1\right)-D_{x}\left(t\right)\\ \; & r_{{}_{t_{n}}}|{}_{P}^{x}=-D_{x}\left(t_{n}\right)\\ \; & r_{t}|{}_{P}^{y}=D_{y}\left(t-1\right)-D_{y}\left(t\right)\\ \; & r_{{}_{t_{n}}}|{}_{P}^{y}=-D_{y}\left(t_{n}\right)\\ \; & r_{t}|{}_{P}^{z}=D_{z}\left(t-1\right)-D_{z}\left(t\right)\\ \; & r_{{}_{t_{n}}}|{}_{P}^{z}=-D_{y}\left(t_{n}\right) \end{array}$$

The expressions of $D_{x}\left(t\right)$, $D_{y}\left(t\right)$ and $D_{z}\left(t\right)$ are as follows.

(17) $$\begin{array}{cc} \; & D_{x}\left(t\right)=\frac{1}{2}{\left(x_{p}\left(t\right)-x_{e}\left(t\right)\right)}^{2}\\ \; & D_{y}\left(t\right)=\frac{1}{2}{\left(y_{p}\left(t\right)-y_{e}\left(t\right)\right)}^{2}\\ \; & D_{z}\left(t\right)=\frac{1}{2}{\left(z_{p}\left(t\right)-z_{e}\left(t\right)\right)}^{2} \end{array}$$

**Figure 3:**
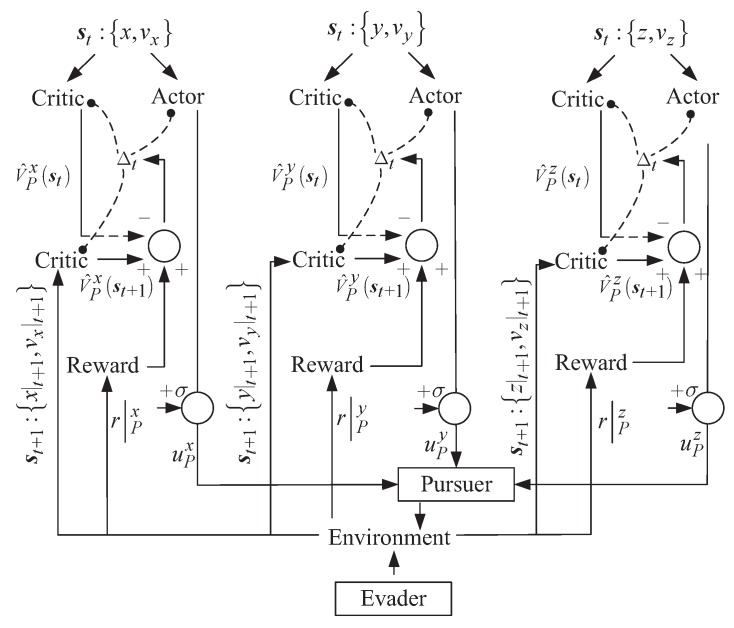
The diagram of the learning logic.

In Figure 3, the learning logic is illustrated. From this figure, it is seen that the learning framework is divided into *x*, *y*, and *z* channels, and each channel has two critic parts and one actor part.

It is noticed that the two critic parts are applied to estimate the value of the current time, $\hat{V}\left(t\right)$, and the value of the next time, $\hat{V}\left(t+1\right)$. It shows that in the *x* channel, the combination of *x* and $v_{x}$ is input into the critic part and the actor part to generate the estimated value ${\hat{V}}_{P}^{x}\left(s_{t}\right)$ and the control variable $u_{P}^{x}$, respectively. Combining $u_{P}^{x}$, $u_{P}^{y}$, and $u_{P}^{z}$, the control vector of the pursuing satellite, $u_{P}$, can be generated. Under such a control policy, the pursuer will interact with the environment, which already contains the motions of the evader. Then, the next state $s_{t+1}$ and the rewards for all the channels are expected to be obtained. Take the *x* channel as an example; the time time difference, $\Delta_{t}$ can be calculated according to $r|{}_{P}^{x}$, ${\hat{V}}_{P}^{x}\left(s_{t}\right)$ and ${\hat{V}}_{P}^{x}\left(s_{t+1}\right)$, and the consequent parameters of the critic part and the actor part can be adaptively tuned through (7) and (10).

### 4.2. Pre-Training Process Based on the Genetic Algorithm

Denote the symbols $\phi_{x}^{P}$, $\phi_{y}^{P}$, and $\phi_{z}^{P}$ as representing the consequent sets of the actor parts in the *x*, *y*, and *z* channels of the pursuer, respectively. The structure of $\phi_{x}^{P}$, $\phi_{y}^{P}$, and $\phi_{z}^{P}$ is defined as a two-dimensional matrix, where the row number depends on the number of membership functions of the first input, and the column number depends on that of the second input. It is supposed that there exist 13 membership functions for the relative distance and 7 membership functions for the relative velocity in each learning channel. Therefore, it is clear that those consequent sets are 13×7 matrices.

Conventionally, the reinforcement learning algorithm is conducted on a totally unknown environment, because the agent is expected to interact with the environment without any external help. However, according to the the human study of orbital dynamics, one can build a mathematical model for the pursuer and the evader in space. Therefore, actually, a part of the real environment seems to be known. To utilize this known part to help find the initial values of the consequent sets, $\phi_{x}^{P}$, $\phi_{y}^{P}$, and $\phi_{z}^{P}$ will be helpful for the learning. Training these consequent sets based on the estimated environment is seen as a pre-training process before the learning.

The known part is defined as an estimated environment, which can obtain the estimated optimal strategy for the pursuer. Denote $\hat{x}={\left[{\hat{x}}_{P},{\hat{x}}_{E}\right]}^{T}$ as the state variable in the estimated environment, where ${\hat{x}}_{P}={\left[x_{p},y_{p},z_{p},v_{p}^{x},v_{p}^{y},v_{p}^{z}\right]}^{T}$ and ${\hat{x}}_{E}={\left[x_{e},y_{e},z_{e},v_{e}^{x},v_{e}^{y},v_{e}^{z}\right]}^{T}$. In addition, denote the estimated ω as $\hat{\omega }$; therefore, the dynamics of the pursuer and the evader in the estimated environment can be expressed as:

(18) $$\dot{\hat{x}}=A\hat{x}+T_{P}B_{P}u_{P}+T_{E}B_{E}u_{E}$$

where:

(19) $$A=\left[\begin{array}{cc} A_{P}\left(t\right) & 0_{6\times 6}\\ 0_{6\times 6} & A_{E}\left(t\right) \end{array}\right]$$

(20) $$A_{P}=A_{E}\left(t\right)=\left[\begin{array}{cccccc} 0 & 0 & 0 & 1 & 0 & 0\\ 0 & 0 & 0 & 0 & 1 & 0\\ 0 & 0 & 0 & 0 & 0 & 1\\ 3{\hat{\omega }}^{2} & 0 & 0 & 0 & 2\hat{\omega } & 0\\ 0 & 0 & 0 & -2\hat{\omega } & 0 & 0\\ 0 & 0 & -{\hat{\omega }}^{2} & 0 & 0 & 0 \end{array}\right]$$

(21) $$B_{P}=\left[\begin{array}{c} 0_{3\times 3}\\ I_{3\times 3}\\ 0_{6\times 3} \end{array}\right]\;B_{E}=\left[\begin{array}{c} 0_{6\times 3}\\ 0_{3\times 3}\\ I_{3\times 3} \end{array}\right]$$

With the cost function, which is shown as follows:

(22) $$J_{i}=D_{i}\left(t_{n}\right)+\int_{t_{0}}^{t_{n}}{\dot{D}}_{i}dt$$

where i=x,y,z, the estimated optimal strategy for the pursuer will be obtained. In this way, the training pairs will be generated, which can be used to train $\phi_{x}^{P}$, $\phi_{y}^{P}$, and $\phi_{z}^{P}$.

**Figure 4:**
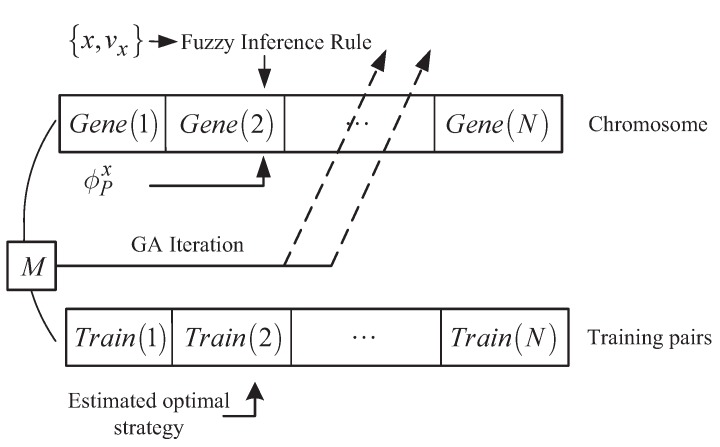
The diagram of the pre-training process.

To approximate the training pairs through the fuzzy inference system, the genetic algorithm (GA) is applied here to conduct the pre-training process. Take the *x* channel as an example. If it is supposed that we can obtain *N* pairs of training data, then the diagram of the GA process is described as in Figure 4.

From the figure, it is seen that the inputs for GA in the *x* channel are *x* and $v_{x}$, which will be input into the fuzzy inference system. The “ chromosome” is a consequent set that is composed of the “genes”. The “genes” are also shown as the consequent parameters. The symbol *M*, which represents the fitness function during the pre-training learning, can be calculated according to the values of $u_{tr}$ from the training data and the values of $u_{A}$ obtained from the fuzzy inference system. The expression of *M* is as below:

(23) $$M=\frac{1}{2}\sum_{i=1}^{N}{\left(u_{A}-u_{tr}\left(i\right)\right)}^{2}$$

where $u_{A}$ is the output of the fuzzy inference system and $u_{tr}\left(i\right)$ is the control value of the ith training pair.

Sorted by the fitness error, the current chromosome will be updated by performing crossover and mutation on the genes. With the help of the GA technique [32], $\phi_{x}^{P}$, $\phi_{y}^{P}$, and $\phi_{z}^{P}$ will be trained to approximate the training data better.

It is noted that the proposed algorithm will make use of the estimated optimal strategy; therefore, the reward function shown in Equation (16) should be consistent with the cost function shown in Equation (22).

## 5. Simulation

A one-to-one space differential game was simulated in this paper. The scenario contained a pursuing satellite *P* and an evading satellite *E*. The reference orbit was a circular orbit with a radius of $6.9\times 10^{3}$ km. Table 1 denote the symbols $x_{P0}$ and $x_{E0}$ as the initial states of the pursuer and the evader, respectively, where the first three items of the vectors represent the position in m and the last three items the velocity in m/s of the agent.

In this scenario, it was supposed that there were some deviations between the real environment and the estimated environment, where the condition $\omega -\hat{\omega }=8\times 10^{-4}$ rad/s existed. In addition, the real environment in this scene was supposed to have the external disturbance item as $d_{t}=\left[1.5\times 10^{-5},1.5\times 10^{-3},2.0\times 10^{-3}\right]$ m/s${}^{2}$. With the learning rate of the critic, α=0.01, the learning rate of the actor β=0.001, the random noise σ=0.1 for exploring, $T_{P}=0.03\times 9.8\times 10^{-3}$ and $T_{E}=0.01\times 9.8\times 10^{-3}$, the proposed PTFRL was processed. As the pursuer and the evader moved in the *x*, *y*, and *z* planes at the same time, the simulation results were drawn in the *X*–*Y* plane and *Y*–*Z* plane, respectively. The total learning process cost 1560 iterations with 3496.98 seconds for learning.

**Figure 5:**
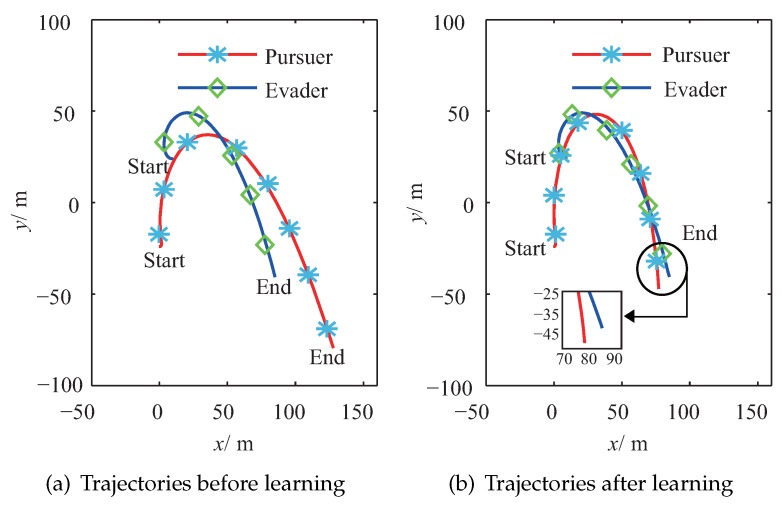
Trajectories of the pursuer and the evader in the *X*–*Y* plane.

**Figure 6:**
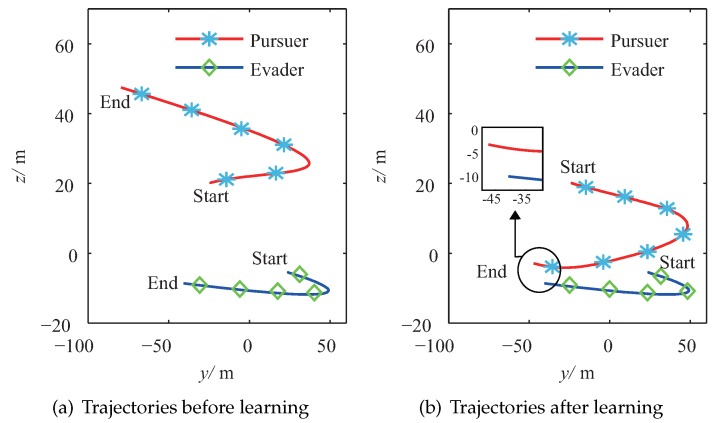
Trajectories of the pursuer and the evader in the *Y*–*Z* plane.

Figure 5a shows the trajectories of the pursuer and the evader after the pre-training process in the *X*–*Y* plane. In this figure, the evader has its optimal strategy, and it is seen that there are some tracking errors from the pursuer to the evader because of the deviations between the estimated environment and the real environment. However, it is seen that the pursuer still has the ability to track the moving trend of the evader because it was pre-trained, and it utilized the information of the estimated environment. Compared with Figure 5a, Figure 5b draws the trajectories of the pursuer and the evader after the proposed PTFRL. It clearly shows that the pursuer could track the evader better after the learning. In the *Y*–*Z* plane, the trajectories before learning and the ones after learning are illustrated in Figure 6a,b, respectively. Due to the largest external disturbance in the *z* channel, Figure 6a shows that the pursuer tracked the evader badly; therefore, there was a big tracking error. In Figure 6b, the pursuer improved its control policy for tracking the evader in the *z* channel. Overall, from Figure 5 and Figure 6, it is shown that, after the proposed learning algorithm, the pursuer could track the evader better because of more suitable consequent set. During the learning process, the pursuer would seek better consequent parameters for different relative states. In this way, the consequent set was updated, which made the pursuer tend to get much closer to the evader.

**Figure 7:**
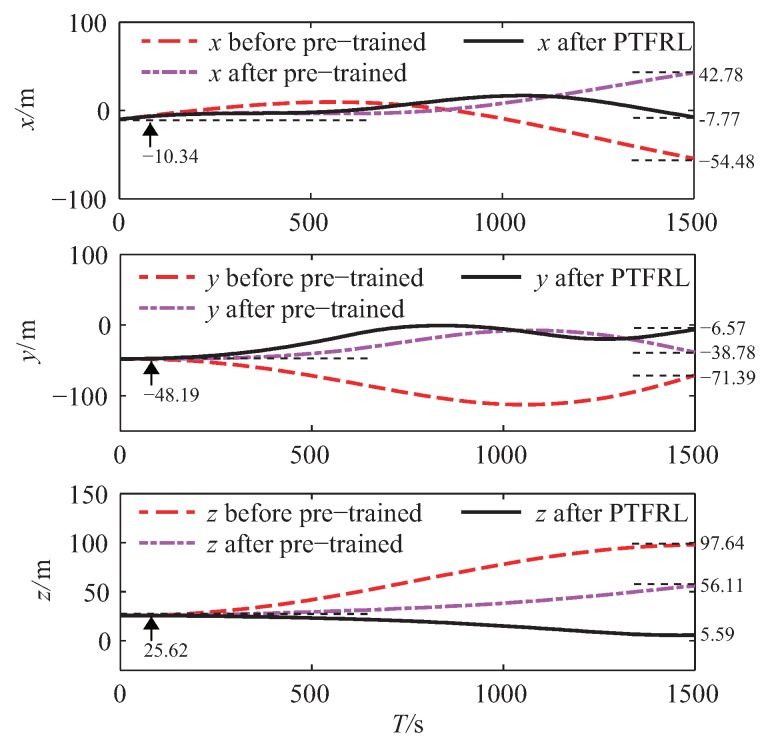
Variations of tracking errors in the *x*, *y*, and *z* channels.

**Figure 8:**
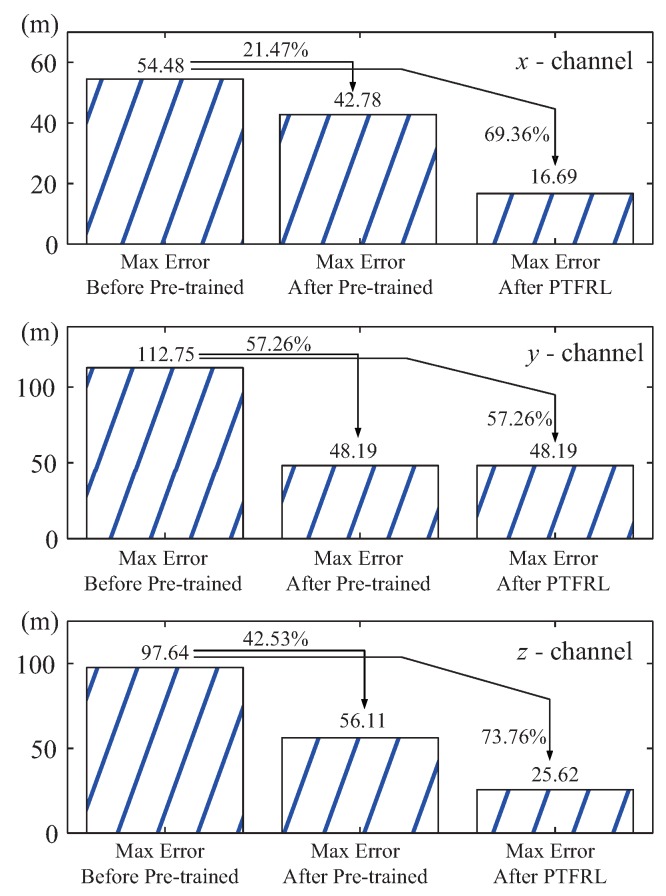
Comparisons of the max tracking errors in different periods.

**Figure 9:**
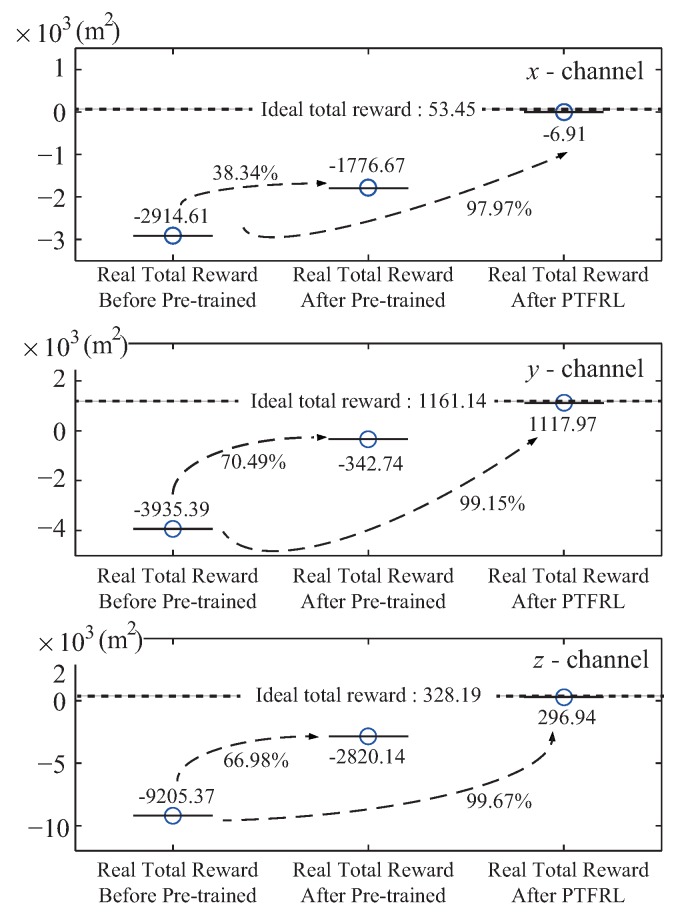
Comparisons of total rewards in different periods.

The whole learning process could be divided into three periods: before pre-training, after pre-training, and after PTFRL. Before pre-training, the pursuer was in free flight without any control policy. When the pursuer finished the pre-training, it took the estimated optimal control policy based on the estimated environment. Finally, when the pursuer took the control policy after PTFRL, this meant that the pursuer finished the learning. The tracking errors under these three periods of the pursuer in the *x*, *y*, and *z* channels are shown in Figure 7. From this figure, it is seen that compared with the tracking error before pre-training, the one after pre-training effectively decreased, and that after PTFRL further approached zero. The max errors under different periods of all channels are drawn in Figure 8. It is clearly seen that, compared with the max error before pre-training, it decreased after pre-training and was further cut down after PTFRL. If all the rewards during the flight were accumulated, the total reward would be obtained. Therefore, there existed the real total reward under the real flight, and the ideal total reward if the pursuer could track the evader perfectly. The ideal total rewards and the real ones in the *x*, *y*, and *z* channels are shown in Figure 9. It shows that the total reward of each channel after pre-training rose compared with that before pre-trained. In addition, the total rewards attempted to approach the ideal values after PTFRL in all channels.

## 6. Discussion

Based on numerical experimental results in Section 5, the following discussions are shown below.

(a) From Figure 7, it can be concluded that in the *x* channel, compared with the terminal tracking error before pre-training, the errors decreased by 21.47% and by 85.74% after pre-training and after PTFRL, respectively. Similarly, the terminal tracking errors decreased by 45.68% and 90.80% after pre-training and after PTFRL in the *y* channel, while the errors decreased by 42.53% and 94.27% after pre-training and after PTFRL in the *z* channel.

(b) In Figure 8, it is seen that, compared with the condition before pre-training, the max tracking error decreased by 21.47% after pre-training, as well as 69.36% after PTFRL in the *x* channel. In the *y* channel, compared with the max tracking error before pre-training, it decreased by 57.26% after pre-training and after PTFRL, because the max error equaled the initial error. Besides, the max error in the *z* channel decreased by 42.53% and by 73.76% after pre-training and after PTFRL, respectively.

(c) Figure 9 shows that if the ideal total reward was set as the target value, the real total reward in the *x* channel improved by 38.34% and by 97.97% after pre-training and after PTFRL, compared with that before pre-training. In addition, the reward improved by 70.49% and 99.15% after pre-training and after PTFRL in the *y* channel. As for the *z* channel, compared with the real total reward before pre-training, the reward improved by 66.98% and 99.67% after pre-training and after PTFRL, respectively.

## 7. Conclusions

To help a pursuer find its advantaged control policy in a one-to-one game in space, an algorithm of pre-trained fuzzy reinforcement learning (PTFRL) was proposed in this paper. To reduce the difficulty of solving without prior information, the man-made model was defined as an estimated environment. By employing the fuzzy inference systems, an actor-critic learning framework, which could be divided into *x*, *y*, and *z* channels, was established. To make use of the estimated optimal strategy, a pre-training process was conducted through initializing the consequent set of the pursuer. With the inputs of the relative position and the relative velocity in each channel, the proposed algorithm controlled the pursuer optimally. By comparing the simulation results before pre-training, after pre-training, and after PTFRL, it was seen that the tracking errors were effectively decreased after the pre-training process and further approached zero after the proposed PTFRL. 

## Figures and Tables

**Table 1:** Initial states of the pursuer and the evader.

State	Value
$x_{P0}$	${\left[-0.422\;m;24.080\;m;20.159\;m;2.678\times 10^{-2}\;m/s;-4.715\times 10^{-5}\;m/s;0\;m/s\right]}^{T}$
$x_{E0}$	${\left[9.918\;m;24.115\;m;-5.462\;m;-2.678\times 10^{-2}\;m/s;-5.608\times 10^{-3}\;m/s;0\;m/s\right]}^{T}$
